# Nature inspired synthesis of magnetite nanoparticle aggregates from natural berthierine†

**DOI:** 10.1039/d3ra04065h

**Published:** 2023-10-31

**Authors:** Alva-Valdivia Luis Manuel, Agarwal Amar, Urrutia-Fucugauchi Jaime, Hernández-Cardona Arnaldo

**Affiliations:** a Laboratorio de Paleomagnetismo, Instituto de Geofísica, Universidad Nacional Autónoma de México Ciudad de México 04510 Mexico; b Department of Earth Sciences, Indian Institute of Technology Kanpur Kanpur 208016 India; c Posgrado en Ciencias de la Tierra, Instituto de Geofísica, Universidad Nacional Autónoma de México Ciudad de México 04510 Mexico

## Abstract

We investigate the origin of the magnetite nanoparticle aggregates (MNAs) from the Peña Colorada iron-ore mining district (Mexico) to devise a nature inspired synthesis process. Three types of samples were used: natural MNAs recovered from the mine, concentrated magnetite microparticles as reference material, and thin berthierine films used to synthesize MNAs. The chemical, mineralogical, crystallographic and rock magnetic properties were determined by polarized microscopy, high-resolution transmission electron microscopy, electron microprobe, X-ray diffraction, Mössbauer spectroscopy, and thermomagnetic and hysteresis measurements. MNAs were synthesized in the lab with the following steps. We start with berthierine thin films, which are heated to temperatures between 495 and 510 °C leading to formation of numerous stable magnetite nanocrystals. They grow at a temperature above 650 °C. Space restrictions lead to the formation of dense MNAs. Smaller MNAs, <200 nm, with a Curie temperature of 650 °C, shows superparamagnetic behavior. While larger MNAs, >7 μm, show Curie temperature of 578 °C and ferromagnetic behavior. Based on present observations, we suggest that MNAs in the Peña Colorada iron-ore formed in a marine environment, where berthierine formation was accelerated by Fe-rich hydrothermal springs that supplied iron and increased the temperature. Most notably, our laboratory experiments mimicked natural conditions and were able to successfully nucleate and grow magnetite nanoparticles which developed into MNAs. These MNAs were similar to those recovered from the Peña Colorada iron-ore deposit. This study, thus, provides a nature inspired method for synthesis of magnetite nanoparticles and its aggregates.

## Abstract 2

We investigate the origin of the magnetite nanoparticle aggregates (MNAs) from the Peña Colorada iron-ore mining district (Mexico) to devise a nature inspired synthesis process.

## Graphical abstract

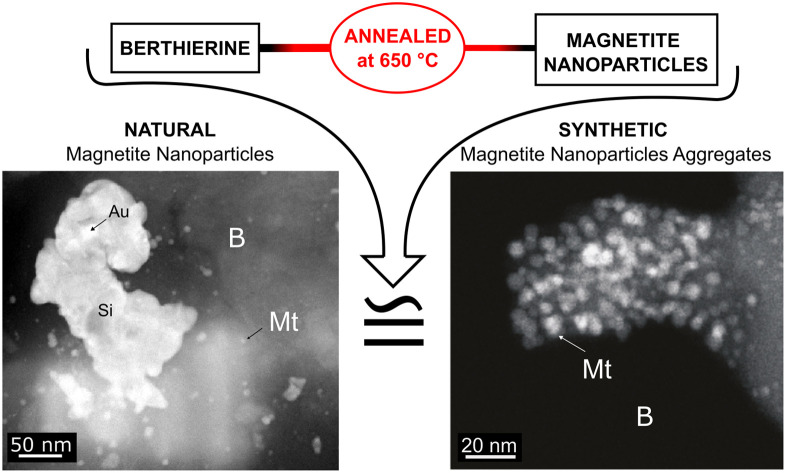

## Introduction

Magnetite nanoparticles and their larger counterparts have contrasting chemical, thermal, optical, physical and electrical properties. While these properties of larger counterparts are well defined, the nanoparticles are still poorly understood.^1–3^ Magnetite nanoparticles are especially relevant due to their applications in chemistry, energy, environment, agriculture and electronics. For example, magnetite nanoparticles have the ability to remediate numerous pollutants, such as heavy metals (lead, chromium, *etc.*), methylene blue, methyl orange, malachite green, and total nitrogen and phosphorus from wastewater, and have antifungal and antibacterial properties.^4–6^

**Fig. 1:**
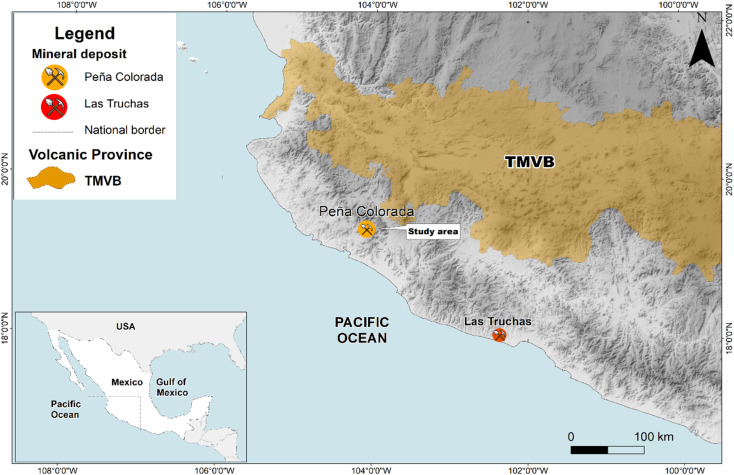
Location of the study area. TMVB: Trans Mexican Volcanic Belt.

Well established methods of synthesizing nanoparticles require hazardous chemicals and physical conditions such as extremely high temperature and pressure. Thus, nature inspired synthesis procedures need to be established and developed.^3,7,8^ To the best of our knowledge such methods for preparing magnetic nanoparticle aggregates (MNAs) have not yet been well established. Our study, thus, investigates the natural origin and genesis of MNAs found in the Peña Colorada iron-ore deposit. This iron-ore mine is located at the Pacific continental margin in southern Mexico (Fig. 1). It is the main iron-ore and pellet producer in Mexico. The main Fe-ore is magnetite, which is being mined since 1975.^9^ We identified natural magnetite nanoparticles that form dense MNAs. Within the MNAs, the open spaces are filled mostly with berthierine, (Fe, Mg, Al)_6_(Si, Al, Fe)_4_O_10_(OH)_8_, and in minor proportion by calcite, quartz, apatite, cryptocrystalline and colloidal silica, hematite, siderite, feldspar, pyrite, chalcopyrite, pyrrhotite, marmatite, galena, covellite, native gold and argentite.^10,11^

**Fig. 2:**
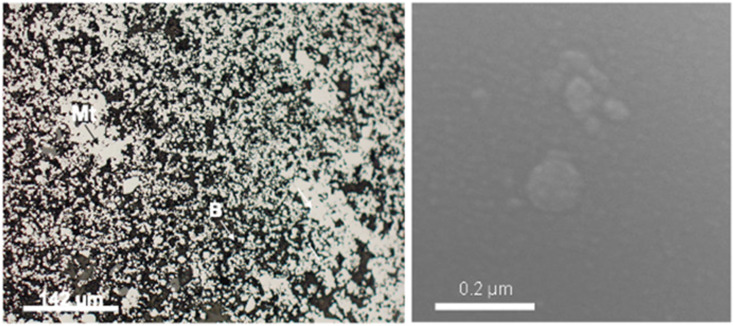
SEM images showing intergranular nanostructured mineral formed by 22 μm to 5 μm MNA embedded in berthierine.

The MNAs were initially reported first during the mine exploitation activities in 1987.^12,13^ In these MNAs, magnetite nanoparticles are embedded in the berthierine matrix (Fig. 2).^2,14,15^ Smaller MNAs, <200 nm, with a Curie temperature of 650 °C, shows superparamagnetic behavior. While larger MNAs, >7 μm, show Curie temperature of 578 °C and ferromagnetic behaviour.^2^ These observations sparked interest in the formation and the magnetic properties of the MNAs, their mineralogical and textural relationships with berthierine, and its implications on the depositional environment.

Recent results^16^ showed a non-linear relation between agglomeration state and magnetic properties. The magnetization in agglomerated magnetite is more sensitive to temperature than the smaller individual dispersed particles.^16–18^ The present study, therefore, investigates the natural MNAs and compares their characteristics with reference magnetite and synthesized MNAs through laboratory experiments. The results underline the use of MNAs as a potential indicator of geological environment in which the iron ore was deposited and, provide a possible nature inspired methodology for preparation of MNAs for societal use.

## Methods and sample description

Three sets of samples were investigated. First were the natural MNAs recovered from the mines. Their microscopic, chemical and rock magnetic properties were studied. Then, these MNAs were heat-treated and again characterized using the same techniques. Second, were standard, 56–30 μm, large magnetite particles in a concentrate. They were used as reference material for the first set. Third, were the MNAs synthesized in lab. These were extracted in the form of berthierine films/lamellae. These films were used for synthesizing MNAs in laboratory conditions that mimic the natural environment during formation. For the characterization and control of the nucleation and growth, we used: optical and stereoscopic microscope, Mössbauer spectroscopy, electron probe X-ray micro-analyzer (EPMA-WDS), differential thermal and gravimetric analysis (DTA-GTA), polarized light microscopy, X-ray diffraction (XRD) and high-resolution transmission electron microscopy.

To study the changes in MNAs with variation in grain size and temperature we used mechanical and magnetic separations. For grain size analysis MNAs were separated into different sizes through a granulometric classification using a Warman-M8 cyclosizer equipment, sub-sieve sizer.^2^ This instrument classifies and separates the particles as a function of the size through centrifugal force under controlled conditions (water temperature, density of the dry sample, flux and time of feeding, *etc.*). MNA fractions were separated into size ranges: 56–30 μm, 30–22 μm, 22–15 μm, 15–10 μm, 10–7 μm and 7–0.1 μm. A few smaller MNAs, 2 to 15 nm in size, were also obtained. Then the effects of temperature on MNAs were analyzed using optical microscopy, differential thermal and gravimetric analysis (DTA-GTA), Xray diffractometry (XRD), electron microprobe (EPMA), and high-resolution transmission electron microscopy (HRTEM). Magnetic characterization was done by analysing the changes in magnetic susceptibility with frequency (*χ*_FD_%) and high-temperature (thermomagnetic), and by investigating the changes in magnetisation with applied field (hysteresis).

Light microscopy was done on a Leica MZ 7.5 stereoscope microscope a Leica DMLP polarizing microscope. XRD was done with Bruker D-8 advance with Cu Kα and *λ* = 1.5418 Å radiation and graphite monochromator. Diffplus B-S software and international crystal powder diffraction (ICPD) database. Electron probe X-ray micro-analyses was done using JEOL JXA 8900-R with dispersive energy spectrometers of X-ray wavelength (WDS) standards SPI#02753-AB. DTA-GTA analyses using a Shimatsu ATR at room conditions with 70 mg sample under temperature from 19 °C to 1100 °C and an interval of 1 °C per minute. HRTEM was done with a JEOL 2010 FEG FASTEM.

*χ*_FD_% and thermomagnetic measurements were done using a Bartington MS2 with an MS2W sensor, coupled to a furnace MS2WFP. Low and high frequency of 470 Hz and 4700 Hz were used for *χ*_FD_% measurements, which are especially useful to distinguish ultrafine magnetite grain with superparamagnetic (SP) behavior. During thermomagnetic measurements the samples were heated in air from room temperature to 700 °C and then cooled back. The magnetic susceptibility is measured during the entire process of heating and cooling. Hysteresis was determined using small core-chips measured in a Princeton Instrument AGFM 2900 (MicroMag) employing a maximum applied field of up to 1.5 T.

## Results

### Natural MNAs

**Fig. 3:**
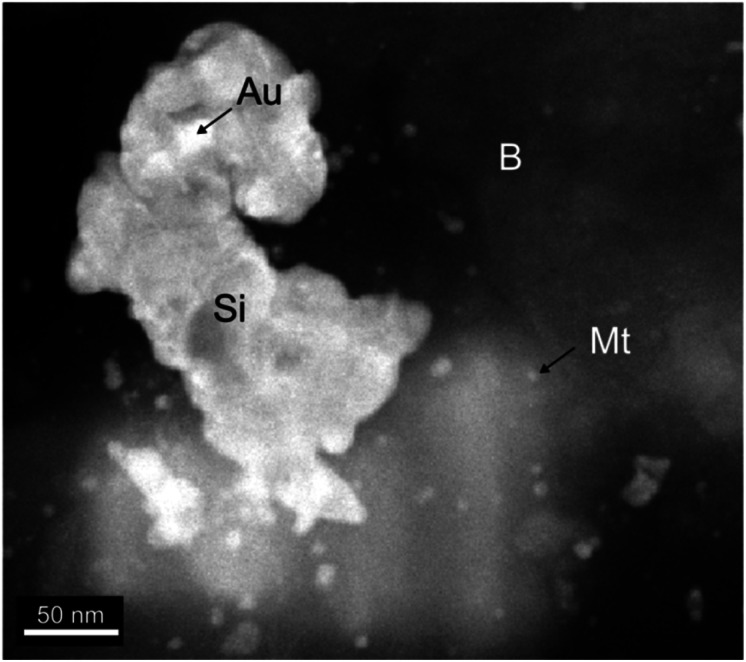
HRTEM of material around the nanoparticles. The spheres of magnetite (Mt) enclosed in berthierine (B) and associated colloidal silica (Si) and gold inclusions (Au).

MNAs are found in the mineralized bodies of the Peña Colorada iron ores. SEM and HRTEM reveal that the MNAs are comprised of spherical magnetite nanoparticles, 2 nm to 15 nm in size (Fig. 2 and 3) and show cubic-octahedra and tetrahedra crystalline structure.^19^ The intergranular material around the nanoparticles contains *ca.* 65% nanoscopic magnetite, *ca.* 30% berthierine, and *ca.* <5% associated minerals, which include, in order of decreasing proportion, granular quartz, amorphous colloidal silica, calcite, siderite, pyrite, chalcopyrite, marmatite, pyrrhotite, feldspar, apatite, sphene, native gold, argentite and organic matter. Berthierine is silky and occurs in low structural order, between amorphous to fine crystalline. The MNAs are occasionally cut by calcite veinlets, up to 20 μm thick, with apatite inclusions. Some MNAs are wrapped by up to a 35 μm thick layer of sulfides.

**Fig. 4:**
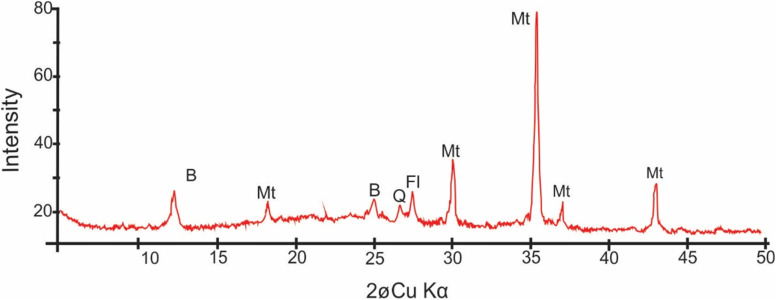
XRD spectrum of the nanoscopic material around the nanoparticles showing characteristic peaks of magnetite (Mt), berthierine (B), feldspar (Fl) and quartz (Q).

The XRD spectrum analysis (Fig. 4) reveals intense magnetite peaks corresponding to (220) *d* = 3.00 Å, (311) *d* = 2.55 Å, (111) *d* = 4.90 and (222) with *d* = 2.43 Å. Berthierine peaks correspond to (001) with *d* = 7.12 Å and (002) with *d* = 3.55 Å. The magnetite peaks are irregularly shaped with wide base, indicating a significant contribution from nanometer-scale grains.^2^

**Fig. 5:**
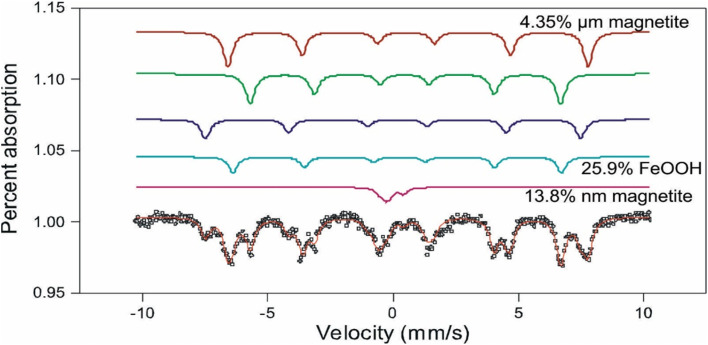
Mössbauer spectrum presenting six spectra doublets, which is typical of magnetite. Two simple unfolds and four doublets correspondent to the magnetite molecule FeO and Fe_2_O_3_ of two oxidation states.

The Mössbauer spectrum of the intergranular material around the nanoparticles (Fig. 5), shows six spectra doublets, typical of magnetite. The Fe_3_O_4_ molecule is in two magnetic states: ferromagnetic state indicated by a sextuple spectrum, corresponding to the micrometric size magnetite aggregates >0.2 μm; and superparamagnetic state represented by a doublet spectrum corresponding to the MNAs of nanometer-scale <200 nm. The magnetic state of the MNAs depends strongly on their degree of compaction.

The chemical properties of the MNAs obtained by WDS multi-elemental analysis in selected fields (ESI Table 1†) reveals Fe^3+^ = 15.59 to 15.69 wt% and Fe^2+^ ions = 7.78 to 7.83 wt%, and elemental trace impurities (<0.2 wt%) of Mn, Ca, Mg, Ti, Al, V, Si, Na and K.

### Laboratory synthesis of magnetite nanoparticles

We performed laboratory experiments, on berthierine thin films, that mimic the natural environment favourable for magnetite nanocrystals and MNA synthesis.

**Fig. 6:**
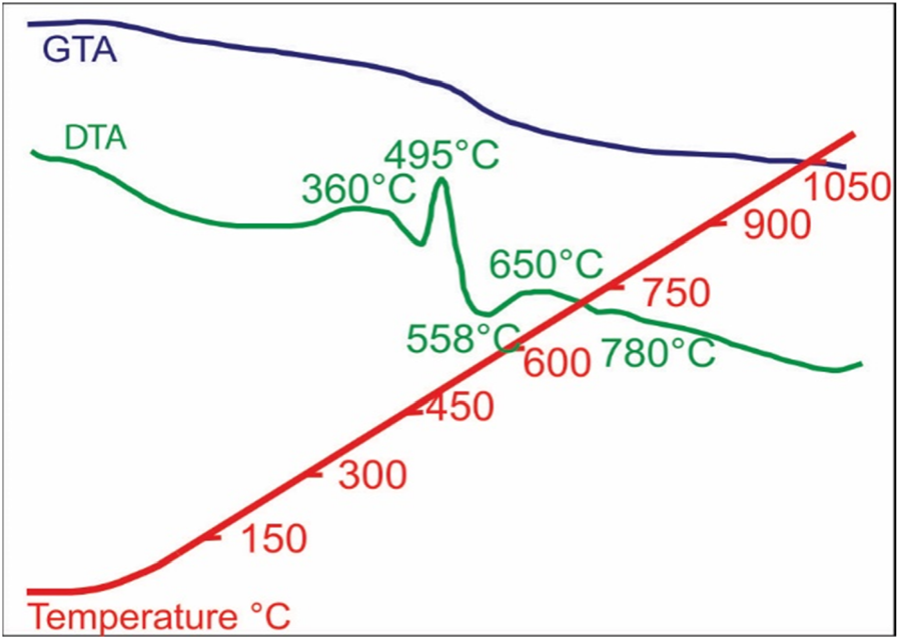
DTA-GTA analysis showing exothermic and endothermic reactions at distinct temperatures, owing to dehydration caused by crystallization, and structural and chemical change (due to magnetite nanoparticles formation). The exothermic reaction at 650 °C, produce magnetite nanoparticles. Later exothermic reaction at 750 °C causes maghemitization and consolidation of these nanoparticles to form MNA.

Thin berthierine films were separated from the ore fragments using a stereomicroscope. The films were annealed at 360, 495, 510, 570, 650 and 750 °C (Fig. 6), and the changes were characterized. Initially at the room temperature, the XRD pattern reveals distinct (001) and (002) peaks of berthierine, which have a *d* value of 7.18 Å and 3.55 Å, respectively. The XRD pattern also presents (311) and (222) magnetite peaks, with *d* value of 2.53 Å and 2.15 Å, respectively.

Exothermic reactions at 360 °C and 495 °C (Fig. 6) mark dehydration of magnetite nanoparticles and berthierine, respectively. Due to the dehydration, at 360 °C, the intensity of (001) and (002) berthierine peaks decreases (Fig. 8). Endothermic dehydration at 430 °C and 550–558 °C (Fig. 6) lead to loss of crystallinity. The berthierine samples, after heating up to 495–550 °C for 2 hours, lose their crystallinity and transform in an amorphous colloid.

**Fig. 7:**
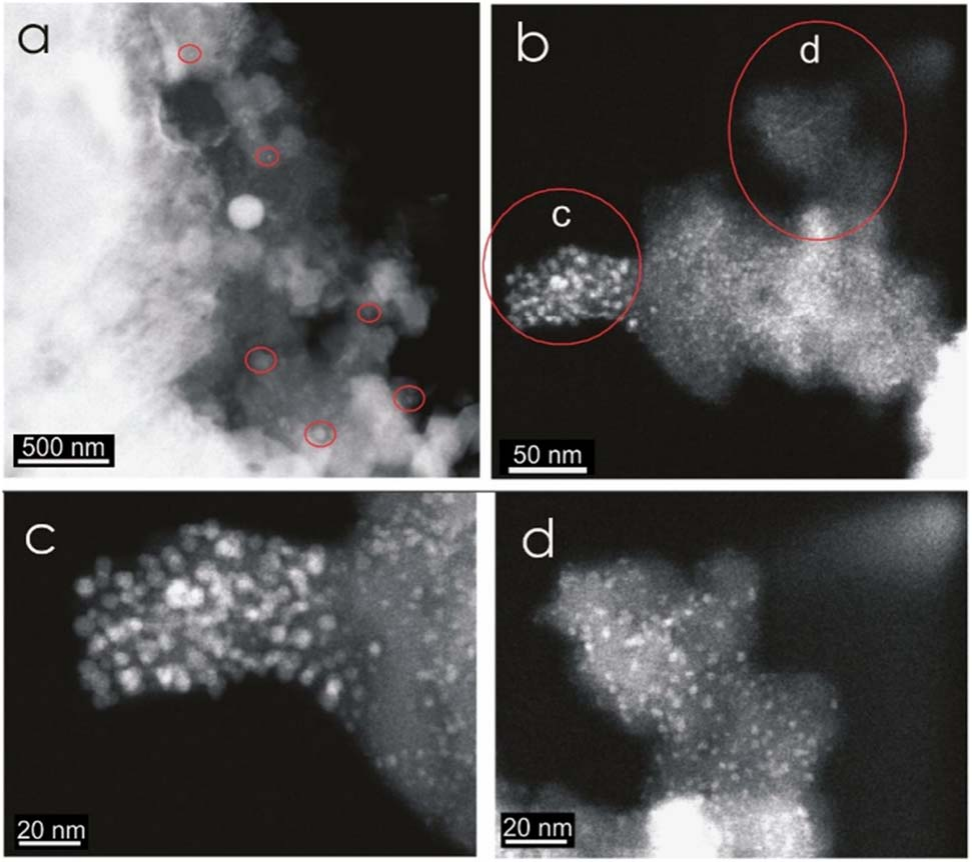
HRTEM contrast *Z* images of a berthierine concentrate annealed at 650 °C. The semispherical magnetite nanoparticles (2 to 255 nm) are embedded in amorphous berthierine matrix.

**Fig. 8:**
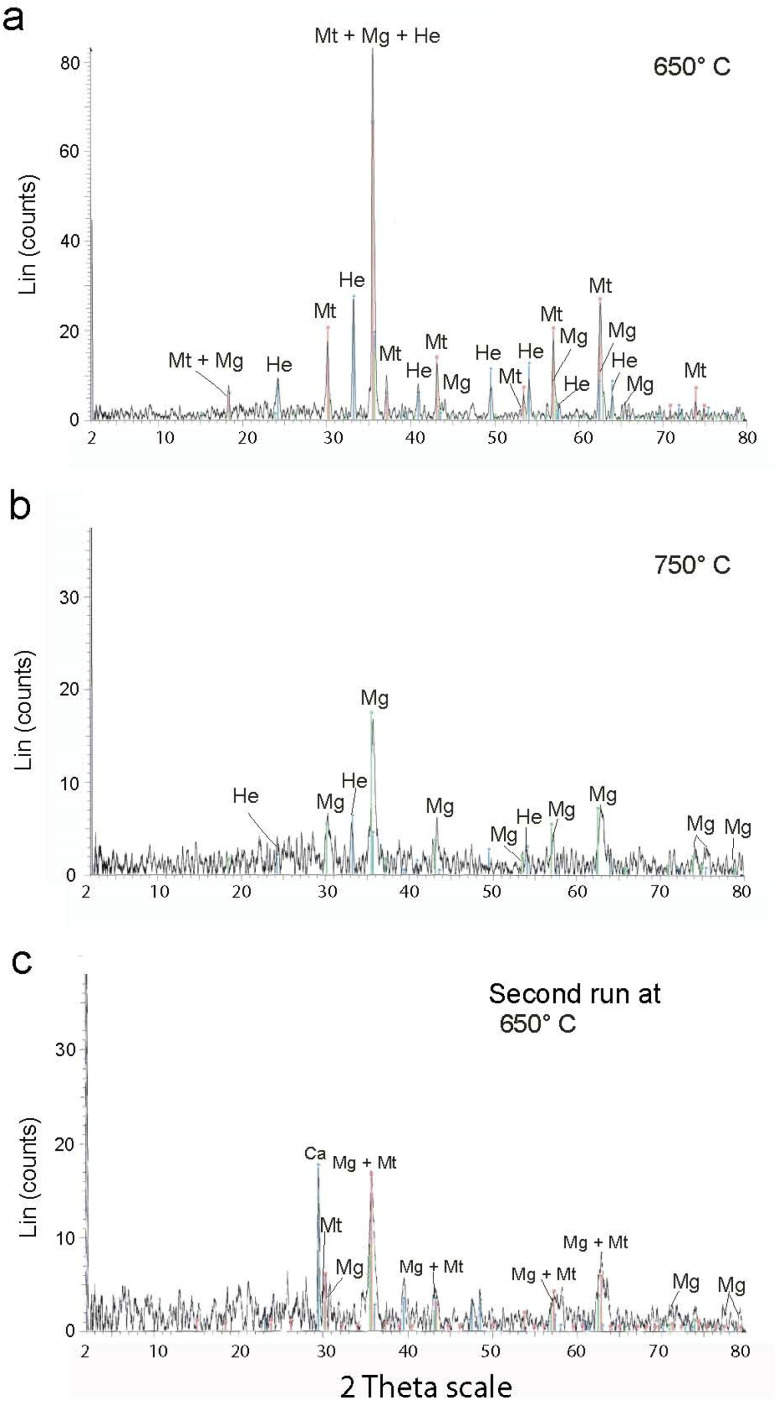
XRD results of MNA synthesis in the laboratory.

**Fig. 9:**
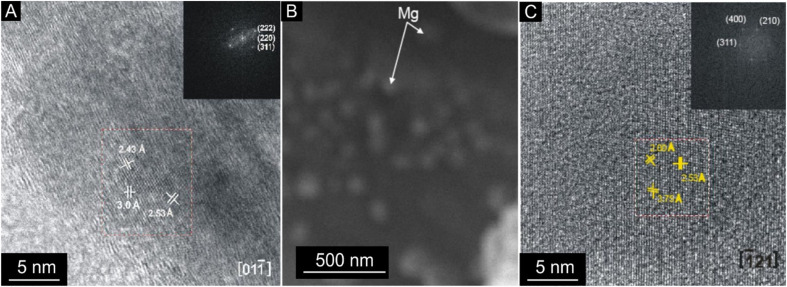
HRTEM analysis (A) magnetite nanoparticle embedded in colloidal berthierine after annealing at 650 °C. It is larger than the base sample before annealing. (B and C) Maghemite nanostructures (Mg) nanostructure after annealing at 750 °C. The observed size is larger than before heating.

Intense exothermic reactions at *ca.* 650 °C is owed to formation of new magnetite nanoparticles and growth of pre-existent magnetite nanoparticles to over 10 nm. These larger nanoparticles saturate the colloid favoring their contact and subsequent aggregation (Fig. 7). New magnetite nanoparticles are represented by novel XRD peaks in the spectra (Fig. 8a) compared to the original (Fig. 4). At 650 °C, the magnetite nanoparticles present zone edge oriented at [011], with interplanar distance *d*1 = 2.43 Å, *d*2 = 2.53 Å and *d*3 = 3.0 Å, and corresponding to (222) (220) and (311) planes, respectively (Fig. 9a). These interplanar distances are consistent with those reported for magnetite.^20^ HRTEM and FFT analyses of these nanoparticles reveals absence of crystal defects and significative structure transformation. This explain their resistance to oxidation and a high *T*_c_.

After the first annealing at 650 °C, we apply second annealing at the same (650 °C) temperature to explore the repeatability of the procedure (ESI Table 2†). XRD reveals similar results (Fig. 8c) and underlines the repeatability of the experiment.

Further annealing at 750 °C leads to oxidation of some magnetite to maghemite. The formation of maghemite is marked by a decrease in intensity of the (311) magnetite peak at 2*θ* angle of 35° (compare Fig. 8a with b). Newly formed maghemite is represented in HRTEM image by crystallites having interplanar distance *d*1 = 2.00 Å, *d*2 = 2.53 Å, *d*3 = 3.79 Å, corresponding to the (400), (311) and (210) planes, respectively (Fig. 9b and c). Magnetic interaction among magnetite nanoparticles results in aggregation of denser magnetite nanoparticles. Exothermic reactions at 750–780 °C mark transformation of magnetite to hematite nanoparticles.

### Effect of temperature on reference magnetite and MNAs

#### Differential thermal and gravimetric analysis

**Fig. 10:**
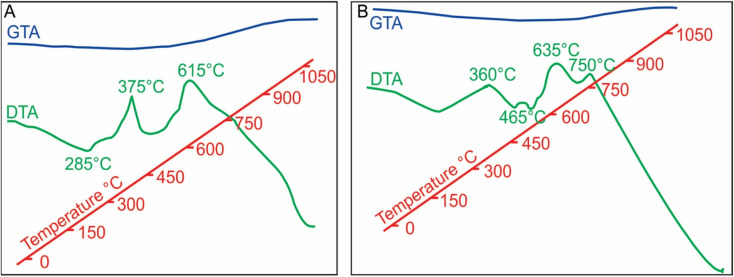
DTA-GTA analysis results: (A) standard magnetite showing two exothermic reactions corresponding to change to maghemite (375 °C) and hematite (615 °C), GTA shows weight increase; (B) MNA show four exothermic reaction corresponding to partial oxidation of superficial to deeper layers (360, 465 and 635 °C) to form maghemite, and afterwards to maghemite (total change) and hematite (at 750 °C), GTA show weight increase after 750 °C due to growth of magnetite nanoparticles.

The DTA and GTA analyses reveal marked differences between reference magnetite, 56–30 μm and natural MNAs recovered from the iron-ores. The reference magnetite presents two exothermic reactions at 375 and 615 °C. The first one corresponds to magnetite changing to maghemite due to oxidation and the second one represents maghemite transforming to hematite (Fig. 10a). The GTA analysis shows a weight loss after 200 °C and weight gain after 615 °C.

In contrast, the MNAs (Fig. 10b) reveal four well defined exothermic reactions at 360 °C (exo-medium), 465 °C (exo-very small), 635 °C (exo-large) and 750 °C (exo-large). The first exothermic reaction, at 360 °C, is owed to partial oxidation and transformation (along the grain boundaries) of magnetite nanoparticles to maghemite (ESI Fig. 1a†). Although the phase change occurs over a wider temperature range 276 to 382 °C, it peaks at 360 °C and is accompanied with 1.14% weight loss. Transformation of magnetite to maghemite is slow process. It requires longer exposers at a specific temperature for oxidation to reach from the crust of the grain to its core. The second, slightly exothermic reaction at 485 °C is marked with a slight bump in the DTA curve. This reaction indicates starting of oxidation MNA core to maghemite.

The high exothermic reactions at 635 °C occur by transformation of the crust of MNAs, formed of maghemite, to hematite (γ-Fe_2_O_3_ → α-Fe_2_O_3_), while their core remains maghemite. The DTA curve shows wide amplitude in the interval 600 to 675 °C (Fig. 10b), supporting the loss of weight as showed in the GTA curve. Optical microscopy and XRD of MNAs annealed at 650 °C reveal a magnetite core, a surrounding layer of maghemite and outermost layer of hematite. The largest MNAs, thus, do not oxidize completely at 650 °C.

In summary, the oxidation front moves slowly towards the interior of the MNAs with gradually increasing temperature from 380 °C to 750 °C (ESI Fig. 1b†). At 750 °C the core of the MNAs are transformed to maghemite, marked by the peak in the DTA-GTA spectrum (Fig. 7b). This is in contrast to the standard magnetite that oxidises to hematite at 615 °C. The oxidation of MNA core at 750 °C is accompanied by a gain in weight of 1.14%. Continued heating till 1100 °C causes complete transformation of MNAs to hematite and a weight gain of 8.18%.

#### Magnetic susceptibility *vs.* frequency measurements

These measurements were done on intergranular material and MNAs annealed at 650 °C, 650 °C (in second run) and 750 °C. The initial *χ*_FD_%, 13%, of natural berthierine concentrate without annealing,^8^ decrease to 7.6% after annealing at 650 °C (ESI Table 3†). This decrease is owed to growth of magnetite nanoparticles leading to the loss of a substantial superparamagnetic fraction.^11^ Further decrease in *χ*_FD_% to 6.5% and 6.7% is seen in sample that was heated to 750 °C and reheated to 650 °C (second run), respectively. This decrease in *χ*_FD_% corresponds to the transformation of remaining fractions of magnetite nanoparticles to maghemite, which present little remanence and very low coercivity (Table 1). In descending order, the coercivity for samples annealed at 650 °C, 750 °C and 650 °C (second run) are 10.23, 7.51 and 5.14 mT, respectively (Table 1, ESI Fig. 2†). After annealing at 750 °C, some magnetite nanoparticles remain unoxidized and the cause of the high saturation magnetization and coercivity in IRM curves (ESI Fig. 3†). Beyond 750 °C magnetite nanoparticles change completely to maghemite. Growth of maghemite leads to decrease of saturation magnetization and coercivity in the IRM curves (ESI Fig. 3†). The hysteresis parameters of the sample annealed to 650 °C and 750 °C show a pseudo-single domain magnetic state.^2,21^

**Table 1:** Hysteresis parameters of a berthierine concentrate after annealed at distinct temperature (*M*, mass; *M*_r_, remanent magnetization; *M*_s_, saturation magnetization; *H*_c_, coercivity; and *H*_cr_, remanence coercivity)

Sample	*M*_r_ (μA m^2^)	*M*_s_ (μA m^2^)	*M*_r_/*M*_s_	*H*_c_ (mT)	*H*_cr_ (mT)	*H*_cr_/*H*_c_	*M* (mg)	*M*_s_/*M* (mA m^2^ kg^−1^)
A-3*n* (base)	0.1549	2.48	0.062	8.18	26.400	3.227	12.0	0.207
A-3*n* annealed at 650 °C	0.0858	7.789	0.011	10.23	24.34	2.38	16.9	0.46
A-3*n* annealed at 750 °C	0.6008	4.285	0.142	7.51	13.59	1.81	21.4	0.2
A-3*n* annealed at 650 °C (2nd run)	0.0341	3.405	0.010	5.14	6.13	1.19	20.3	0.17

#### Thermomagnetic measurements

**Fig. 11:**
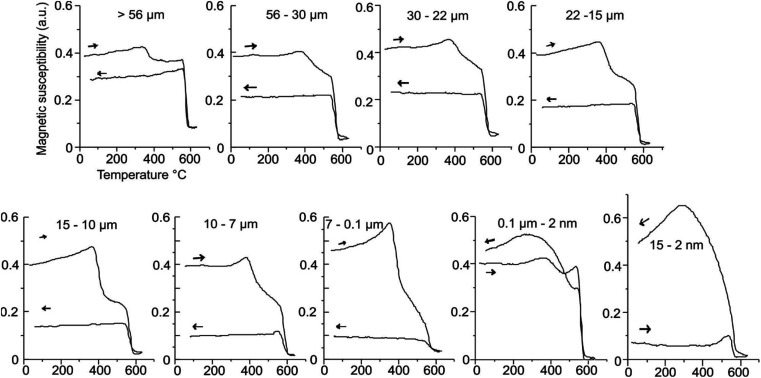
Magnetic susceptibility *vs.* high temperature curves of MNAs in distinct range sizes.

Heating curves of MNAs do not show any remarkable change in susceptibility up to 350 °C (Fig. 11). The first Curie temperature (*T*_c_) is encountered at 350–380 °C indicating maghemite,^22,28,29^ which is produced from the transformation of magnetite nanoparticles.^23^ All MNA sample show moderate to strong Hopkinson peak at *c*. 580 °C. Hopkinson peak can be attributed to abundance of smaller magnetite grains. At temperatures above the magnetite *T*_c_, the thermomagnetic curves are reversible with the colling branch presenting the same shape as the heating branch (Fig. 11). Between magnetite *T*_c_ and room temperature, the cooling branch of the thermomagnetic curves presents lower magnetic susceptibility than the heating branch. Notably, the samples with smaller magnetite grain size show lower average magnetic susceptibility (Fig. 11). The smaller magnetite grains are unoxidized cores of the larger MNAs.

MNA samples with largest grain size, 56 to 19 μm, annealed at 380 °C, have maghemite at boundaries with magnetite at the core (Fig. 1a, ESI†). With increasing annealing temperature, maghemite proportions increase. This is evident in the heating branch of thermomagnetic curves, where the maghemite *T*_c_, between 350–380 °C becomes more prominent with the with increasing heat treatment (Fig. 11).

## Discussion

We start by studying temperature-induced mineral phase changes in MNAs. We designed experiments to produce synthetic MNAs. Previous reports,^12,13,24–27^ on magnetite nanocrystals were used as reference for this study.

### Laboratory synthesis of magnetite nanoparticles

We develop an experiment to mimic natural conditions and synthesize MNAs. The mineralogical changes at each step of the experiment were quantified by DTA-GTA, XRD, HRTEM, and magnetic measurements. We started with extracting the berthierine films from natural iron ore. The films were then heated. Berthierine starts to dehydrate at 360 °C without significant changes. At 495 °C changes initiate in berthierine with further dehydration and the structural transformation to an amorphous colloid. This transformation is complete at 550–558 °C. At 635 °C, new magnetite nanoparticles form, while pre-existing magnetite nanoparticles grow rapidly. At 650 °C, magnetite nanoparticles continue to grow beyond 10 nm. These nanoparticles saturate the colloid, favouring the contact and aggregation to form MNAs. Another annealing at 650 °C, done to check the reproducibility, presents similar results (ESI Table 2†). With further heating to higher temperature of 750 °C, some magnetite in the MNAs starts to oxidize to maghemite along the boundaries. At 780 °C the oxidation along the outer boundaries is complete leading to development of hematite.

This laboratory synthesis is analogous to enrichment of berthierine in marine environment, where, colloidal berthierine is constantly enriched with Fe from external sources such as marine chimneys that discharge Fe-rich brines. The increasing of temperature during the experiment mimics the temperature increase due to the hydrothermal pulses and works in favor of nucleation and growth of magnetite nanoparticles, which later form MNAs.

### Effect of temperature on the reference magnetite and natural MNAs

DTA-GTA analysis highlights the differences in the behaviour of natural MNAs from the Peña Colorada (Fig. 10b) with respect to the reference material, concentrate of 56–30 μm size magnetite crystals (Fig. 10a).

Firstly, the reference magnetite shows three well-defined exothermic reactions at 285 °C, 375 °C and 615 °C (Fig. 10a) during its complete change to maghemite and later to hematite, Fe_3_O_4_ → γ-Fe_2_O_3_ → α-Fe_2_O_3_. On the contrary, the MNAs reveal four well-defined exothermic reactions at 360 °C, 485 °C, 635 °C, 750 °C (Fig. 7b). The first two correspond to partial oxidation to maghemite and hematite, respectively, and the last two reactions (465 °C and 750 °C), perhaps to the complete oxidation. The MNAs start to lose weight from 200 °C to 727 °C and thereafter gain weight due to the growth of magnetite nanoparticles up to 1050 °C.

Secondly, the reference material presents a Tc of 580 °C, which agrees with the published Curie point for pure magnetite.^30^ Whereas, Tc of the MNAs is >650 °C, which may due to hematite.

To summarize, the thermal reactions in reference magnetite initiate at lower temperatures and progress rapidly, while in the MNAs thermal reactions initiate at higher temperatures and progress slowly according, demonstrating the heat resistance of MNAs.

### Implications on the genesis of MNAs in nature

The synthesis of MNAs takes three steps: (1) nucleation and growth of numerous stable crystalline magnetite nanoparticles homogeneously distributed in a colloidal berthierine matrix starts at 635 °C. (2) With increasing temperature, to 650 °C, the nucleation accelerates, and the pre-existing nanoparticles grow. HRTEM confirms that these particles are magnetite less than 15 nm. (3) Constant temperatures of 650 °C lead to the continuous growth of magnetite nanoparticles. Space restrictions lead to the formation of dense MNAs less than 200 nm in size.

In marine conditions, berthierine is formed by colloidal diagenetic processes. Supply of heat and iron from hydrothermal solutions coming out of marine chimneys^31^ could spark a series of reactions and physicochemical adjustments in composition to form the MNAs.

## Conclusions

The properties of MNAs are size-dependent. Magnetite MNAs, which are larger than 7 μm, start to transform to maghemite in the range of 280 °C to 380 °C and then to hematite at 615 °C. In contrast, the smaller MNAs, <0.2 μm, are more resistant to temperature induced oxidation and transformation. They show changes to hematite at temperatures >750 °C. The larger MNAs are ferromagnetic, while the smaller ones are superparamagnetic.

Most notably, our laboratory experiments that mimicked natural conditions and were able to successfully nucleate and grow magnetite nanoparticles which developed into MNAs. The MNAs were similar to those recovered from the Peña Colorada iron-ore deposit. This study provides a nature inspired method for synthesis of magnetite nanoparticles and its aggregates which do not require harmful chemicals and the starting material is also naturally derived.

The physical and chemical settings of the experiments allow the prediction of the environmental conditions for the formation of the natural magnetite nanoparticle aggregates. It is suggested that the magnetic nanoparticle aggregates at the Peña Colorada iron mine developed in shallow to a deep marine environment with redox conditions. This was facilitated by Fe rich hydrothermal source such as marine chimneys. Because of this, we propose that the MNAs act as genetic indicators and reveal the geological conditions of their formation.

## Author contributions

Alva-Valdivia: conceptualization, formal analysis, funding acquisition, writing – original draft, supervision. Agarwal: methodology, data curation, writing – review&editing. Urrutia-Fucugauchi: writing – review&editing, Hernández-Cardona: writing – review & editing.

## Conflicts of interest

There are no conflicts to declare.

## Supplementary Material

RA-013-D3RA04065H-s001
