# Supplementary material for: Nature inspired synthesis of magnetite nanoparticle aggregates from natural berthierine
Source: RSC Adv. 2023 Oct 31;13(46):32054–62. doi: 10.1039/d3ra04065h (PMC10617585; doi:10.1039/d3ra04065h)
Supplement: RA-013-D3RA04065H-s001 [file RA-013-D3RA04065H-s001.pdf]

Electronic Supplementary Material (ESI) for RSC Advances.
This journal is © The Royal Society of Chemistry 2023

Supplementary file for:

Characterization of natural magnetite nanoparticles aggregates from the Peña Colorada iron ore deposit, Mexico: genetic inferences

Alva-Valdivia, L. M. (1), Agarwal A. (2), Urrutia-Fucugauchi, J. (1) and Hernández-Cardona, A. (3)

(1) Laboratorio de Paleomagnetismo y Geofísica Nuclear, Instituto de Geofísica, Universidad Nacional Autónoma de México, México (DF) 04510 México.

(2) Department of Earth Sciences, Indian Institute of Technology Kanpur, Kanpur, 208016, India

(3) Posgrado en Ciencias de la Tierra, Instituto de Geofísica, Universidad Nacional Autónoma de México, Ciudad de México, 04510, México.

Supplementary Table 1. Chemical composition and structural formula of the MNA (intergranular mineral; Mt, magnetite; B, berthierine; quartz, Q; calcite, C; and feldspar, Fl).

	PCA - 1	*PCA - 2*	*PCA - 3*
Fe_2O_3	62.299	62.310	61.580
FeO	27.987	27.994	27.664
TiO_2	0.584	0.234	0.224
MnO	0.212	0.043	0.033
NiO	0.044	----	0.015
Cr_2O_3	-----	0.024	0.024
CoO	0.118	0.144	0.101
V_2O_3	0.163	0.160	0.156
MgO	0.297	0.037	0.088
CaO	0.005	0.018	0.199
SiO_2	0.057	0.042	0.052
Al_2O_3	0.379	0.399	0.417
Na_2O	0.065	0.102	-----
K_2O	-----	0.014	-----
**Σ**	*92.210*	*91.512*	*90.553*
Fe_{+3}	15.593	15.667	15.690
Fe_{+2}	7.786	7.821	7.833
Ti	0.146	0.058	0.057
Mn	0.559	0.012	0.010
Ni	0.012	-----	0.004
Cr	-----	0.008	0.008
Co	0.032	0.038	0.026
V	0.043	0.044	0.041
Mg	0.139	0.018	0.045
Ca	0.018	0.006	0.071
Si	0.018	0.014	0.018
Al	0.099	0.157	0.167
Na	0.039	0.064	-----
K	-----	0.004	-----
**Σ**	*24.484*	*23.911*	*23.970*

Supplementary Table 2. XRD analyses of MNA after annealing at 650 °C and 750 °C, and 650 °C (second run).

**Mineral**	**Concentrated MNA**	**Annealed at 650°C**	**Annealed at 750°C**	**Annealed at 650°C (second round)**
Magnetite	x	x		x
Maghemite		x	x	x
Hematite		x	x	x

Supplementary Table 3. Magnetic susceptibility vs. frequency measurements during laboratory synthesis of magnetite nanoparticles after annealing at 650 °C, 750 °C and 650 °C (second run).

Sample	Magnetite nanoparticles	χLF 10-6 m3 Kg-1	χHF 10-6 m3 Kg-1	χFD 10-6 m3 Kg-1	χFD%
PC7	~ 2-8 nm	3.31	2.88	0.43	13.0
PC7 650 °C	~ 2-8 nm	61.75	57.05	4.70	7.6
PC7 750 °C	~ 2-8 nm	46.91	43.86	3.05	6.5
PC7 650 °C (second heating)	~ 2-8 nm	11.20	10.45	0.75	6.7

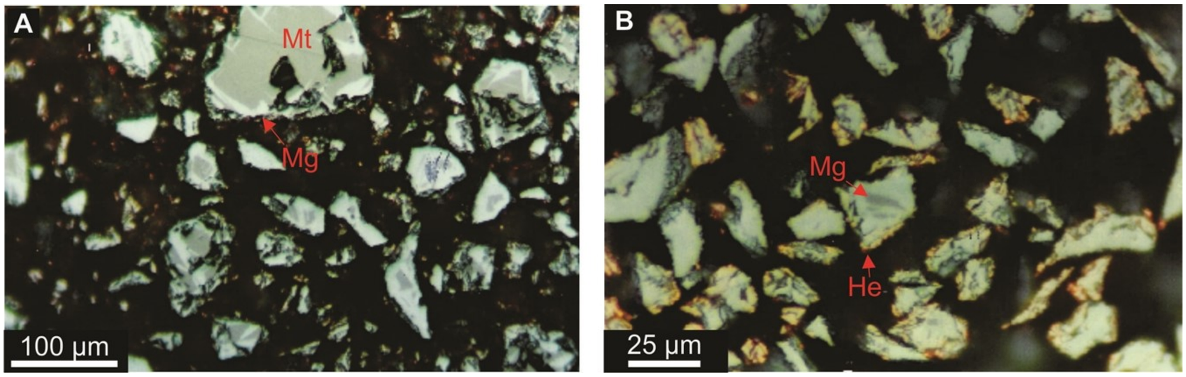

Supplementary Figure 1. Reflected light images presenting (a) MNAs annealed at 360 °C with a crust of maghemite (Mg) and magnetite (Mt) cores, and (b) MNAs annealed at 750 °C with crust of hematite (He) and maghemite (Mg) cores.

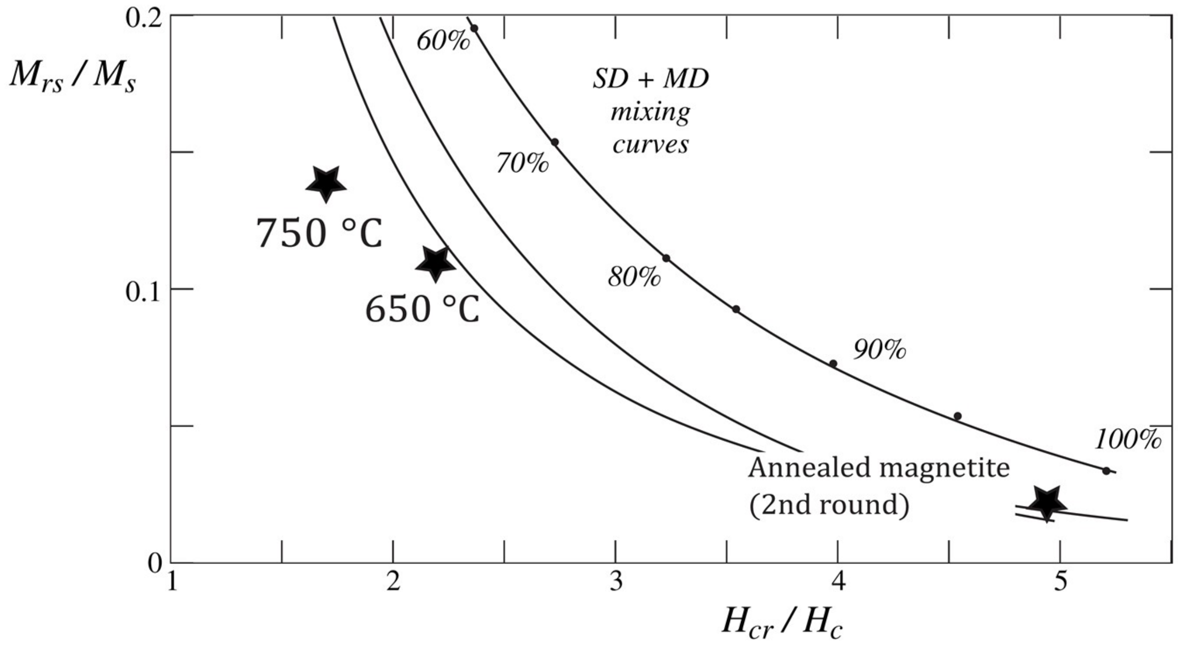

Supplementary Figure 2. Hysteresis parameter ratio plot (coercivity, Hcr/Hc and magnetization, Mr/Ms) of berthierine concentrate sample after annealing (Day et al, 1977).

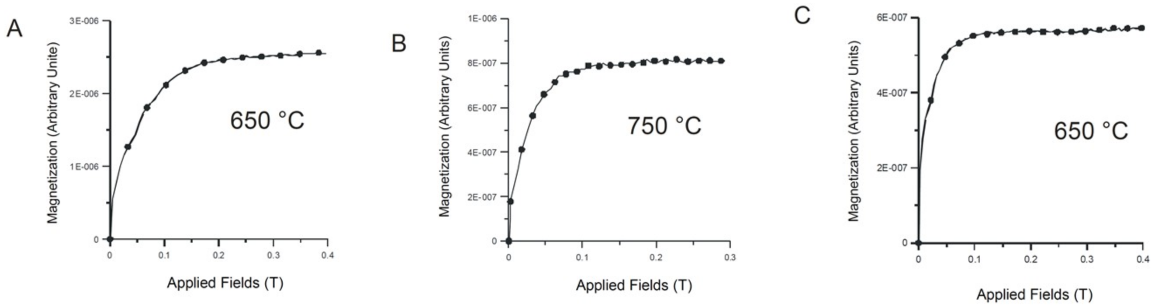

Supplementary Figure 3. IRM results of berthierine concentrate samples after annealing.
